# Bach Flower Remedies for psychological problems and pain: a systematic review

**DOI:** 10.1186/1472-6882-9-16

**Published:** 2009-05-26

**Authors:** Kylie Thaler, Angela Kaminski, Andrea Chapman, Tessa Langley, Gerald Gartlehner

**Affiliations:** 1 Department for Evidence-based Medicine and Clinical Epidemiology, Danube University, Krems, A-3500, Austria; 2 Ludwig Boltzmann Institute for Health Technology Assessment, Garnisongasse 7/20, A-1090 Vienna, Austria

## Abstract

**Background:**

Bach Flower Remedies are thought to help balance emotional state and are commonly recommended by practitioners for psychological problems and pain. We assessed whether Bach Flower Remedies (BFRs) are safe and efficacious for these indications by performing a systematic review of the literature.

**Methods:**

We searched MEDLINE^®^, Embase, AMED, and the Cochrane Library from inception until June 2008 and performed a hand-search of references from relevant key articles. For efficacy, we included all prospective studies with a control group. For safety, we also included retrospective, observational studies with more than 30 subjects. Two authors abstracted data and determined risk of bias using a recognised rating system of trial quality.

**Results:**

Four randomised controlled trials (RCTs) and two additional retrospective, observational studies were identified and included in the review. Three RCTs of BFRs for students with examination anxiety, and one RCT of BFRs for children with attention-deficit hyperactivity disorder (ADHD) showed no overall benefit in comparison to placebo. Due to the number and quality of the studies the strength of the evidence is low or very low. We did not find any controlled prospective studies regarding the efficacy of BFRs for pain. Only four of the six studies included for safety explicitly reported adverse events.

**Conclusion:**

Most of the available evidence regarding the efficacy and safety of BFRs has a high risk of bias. We conclude that, based on the reported adverse events in these six trials, BFRs are probably safe. Few controlled prospective trials of BFRs for psychological problems and pain exist. Our analysis of the four controlled trials of BFRs for examination anxiety and ADHD indicates that there is no evidence of benefit compared with a placebo intervention.

## Background

Bach flower remedies (BFRs) are a widely-available, popular form of complementary and alternative medicine (CAM) developed in the 1930s by the British physician Dr Edward Bach. Bach devoted his life to the discovery of 38 remedies that correspond to 38 negative emotional states (Table 1). [1,2] Bach believed in a truly holistic form of emotional healing; BFRs are believed to assist the body in healing itself by providing "a positive emotional state that is conducive to the restoration of a healthy equilibrium and by acting to catalyze an individual's own internal resources for maintaining balance".[3] According to Bach, the restoration of balance could be used for treating any medical condition, however BFRs are commonly used for psychological problems and stress.[4]

**Table 1:** The Bach flower remedies and their indications

*Agrimony*	mental torture behind a cheerful face
*Aspen*	fear of unknown things
*Beech*	Intolerance, perfectionist
*Centazry*	the inability to say 'no'
*Cerato*	lack of trust in one's own decisions
*Cherry Plum*	fear of the mind giving way, fear of losing control
*Chestnut Bud*	failure to learn from mistakes
*Chicory*	selfish, possessive love, needs the appreciation of others
*Clematis*	dreaming of the future without working in the present, absentminded
*Crab Apple*	the cleansing remedy, also for self-hatred, poor body image
*Elm*	overwhelmed by responsibility, pressures of work
*Gentian*	discouragement after a setback, pessimism
*Gorse*	hopelessness and despair
*Heather*	self-centredness and self-concern
*Holly*	hatred, envy and jealousy, feels victimized
*Honeysuckle*	living in the past, overwhelming nostalgia for the past
*Hornbeam*	procrastination, tiredness at the thought of doing something
*Impatiens*	impatience
*Larch*	lack of confidence, competent but fear failure
*Mimulus*	fear of known things, shy, nervous personality
*Mustard*	deep gloom for no reason
*Oak*	the plodder who keeps going past the point of exhaustion
*Olive*	exhaustion following mental or physical effort
*Pine*	guilt, self-blame
*Red Chestnut*	over-concern for the welfare of loved ones
*Rock Rose*	terror and fright, useful for nightmares
*Rock Water*	self-denial, rigidity and self-repression
*Scleranthus*	inability to choose between alternatives
*Star of Bethlehem*	Shock, loss, bereavement, trauma
*Sweet Chestnut*	Extreme mental anguish, when everything has been tried and there is no light left
*Vervain*	over-enthusiasm, perfectionism
*Vine*	dominance and inflexibility
*Walnut*	protection from change and unwanted influences, birth, puberty, divorce
*Water Violet*	pride and aloofness
*White Chestnut*	unwanted thoughts and mental arguments, unwanted thoughts, unable to concentrate
*Wild Oat*	uncertainty over one's direction in life
*Wild Rose*	drifting, resignation, apathy
*Willow*	self-pity and resentment
*Rescue Remedy*	Composite remedy consisting of Star of Bethlehem, Rock Rose, Cherry Plum and Clementis; for emergencies to combat fear, panic, shock and fear of losing control

Source: [1,5]

Only flowers that grow naturally in the wild are suitable for preparation. BFRs are prepared in two ways following Bach's precise directions: the sun method and the boiling method. In the sun method, fully opened flower heads still fresh with dew are floated on the surface of pure spring water in a glass bowl and left for a few hours in the sunshine, whereas in the boiling method, used for trees and bushes, the branches and leaves are boiled in water for half an hour.[1] In both methods, the plant matter is removed, and, according to Bach, the water retains the vibrations or energy of the flower. The liquid, called the mother tincture, is filtered and mixed with brandy, which acts as a preservative.[5] The remedies can be taken orally diluted in a glass of water, or applied directly to pulse points such as the wrists, temples and behind the ears.[3] They can be used individually or in combination with up to seven other tinctures.[5] BFRs do not contain pharmacologically relevant remnants of the original flowers[6] and are considered safe to use in combination with other medications, as well as by pregnant women, children, babies and the elderly.[5,7] However, it has been suggested that BFRs could be dangerous for recovering alcoholics due to their alcohol content.[4]

Bach's 38 flower remedies each address one of the seven psychological causes of illness: fear, uncertainty, insufficient interest in present circumstances, loneliness, oversensitivity to influences and ideas, despondency or despair and overcare for the welfare of others.[7] The individual patient is prescribed particular remedies depending on the acute problem at hand, which should be individually tailored and adjusted during the course of therapy, typically over weeks to months.[1,7] For example, the flower 'impatiens' is used for impatience and irritability, 'mimulus' for fear of known things, shyness, and timidity, and 'olive' for those that are drained of energy.[7] In addition, some BFRs are categorized as "type" remedies and are specific to a certain character trait or disposition.[1] A person who suffers from overwhelming guilt might be offered pine as a type remedy, and chronically indecisive people could benefit from Scleranthus.[1] Three BFRs are helpful to unblock the energy flow in patients without obvious symptoms: Wild Oat, Holly, and Star of Bethlehem.[7] Because the relief of anxiety is a major factor in pain relief, proponents of BFRs have suggested that BFRs also have the potential to function as a therapeutic agent for pain.[8] "Rescue Remedy", also known as "Five Flower Remedy", is the only combination of BFRs determined by Bach himself and functions as an all purpose emergency agent in situations of acute anxiety or distress. It contains a mixture of star of Bethlehem (Ornithogalum umbellatum), rock rose (Helianthemum nummularium), impatiens (Impatiens glandulifera), cherry plum (Prunus cerasifera), and clematis (Clematis vitalba). [1,3,5,7] Rescue remedy is recommended as a first aid preparation for situations where acute stress is likely to occur.

According to Bach, the remedies work through the life force energy or vibration that is transmitted from the flowers to the tincture. This vibration interacts on a subtle energy level with the individual to rebalance the conscious and unconscious and dissolve old patterns of behaviour.[5] By alleviating negative feelings and relieving the underlying emotional and psychological problems of the patient, a physical healing is enabled. Patients sometimes experience a worsening of their symptoms before an improvement, which can manifest as aggravation.[9]

Training as a Bach Flower Remedy practitioner is offered through the Bach Foundation in the UK and at other centres in Europe.[10,11] The courses are designed for therapists in similar fields who would like to incorporate BFRs into their practice. BFRs are also available "over-the-counter "in some countries, and on-line through several websites.[12] One bottle of BFRs cost approximately £6 (€7 or US$10).

A previous systematic review of BFRs for overdue birth, examination anxiety, and depression concluded that the available studies did not indicate that BFRs are clinically different to placebo for those indications.[6] Our review was initially undertaken to provide evidence-based guidance to an Austrian state insurance agency regarding financing complementary and alternative therapies. The objective of our study was to examine the evidence on both the efficacy and safety of BFRs. For this reason we included evidence from controlled trials as well as observational studies. We limited indications of interest to psychological problems and pain because these are the most common indications for BFRs. [8,13]

## Methods

### Literature search

To identify relevant studies we searched MEDLINE^®^, Embase, AMED, and the Cochrane Library up to June 2008, covering the entire time span of the databases. We used either Medical Subject Headings (MeSH) as search terms when available or key words when appropriate. We searched for variations of the terms "Bach flower remedies", "flower essence", "Bach flower", "rescue remedy" and "flower therapy" [see Additional file 1]. Other flower essence treatments exist that were not described by Bach, [14] however, BFR practitioners do not advocate their use.[10] Therefore, we limited our review to the 38 BFRs plus Rescue Remedy. We limited searches to English and German language literature, which reflects the language capabilities of our team. In addition, we manually searched reference lists of pertinent review articles and letters to the editor to identify additional evidence.

### Study selection

Two persons independently reviewed abstracts and relevant full-text articles. To assess efficacy regarding outcomes of interest, we included randomized controlled trials as well as prospective, controlled observational studies. To determine the risk for harms (specific adverse events, rates of adverse events, and discontinuations attributable to adverse events), we also included data from uncontrolled and retrospective observational studies with ≥ 30 participants. Table 2 summarizes the eligibility criteria.

**Table 2:** Eligibility criteria

**Population**	Patients with pain and/or psychological symptoms
**Intervention**	Bach flower remedies only

**Comparison**	Placebo

**Outcomes**	Pain reduction, improvement of symptoms, adverse events

**Timing**	No restriction

**Setting**	No restriction

**Study design**	For efficacy: all prospective, controlled studies; no sample size limitations For harms: all prospective, controlled studies and all observational studies with a minimum sample size of 30.

If both reviewers agreed that a study did not meet eligibility criteria, we excluded it. We also formally excluded studies that met eligibility criteria but were reported only as an abstract or as a letter to the editor. Investigators resolved disagreements about inclusion or exclusion by consensus or by involving a third reviewer.

### Data extraction and assessment of risk of bias

Trained reviewers abstracted data from each study and assigned a quality rating. A senior reviewer read each abstracted article, evaluated completeness of data abstraction, and confirmed the quality rating. Investigators resolved any disagreements by discussion and consensus or by consulting an independent party.

We assessed the risk of bias of trials based on the Cochrane Collaboration's tool for assessing risk of bias and applied ratings of high, unclear or low. [15] Primary elements of quality assessment for trials included randomization sequence generation and allocation concealment, similarity of compared groups at baseline, blinding, completeness of outcome data and outcome reporting, and overall and differential loss to follow-up. To assess observational studies, we used criteria involving selection of cases or cohorts and controls, adjustment for confounders, methods of outcomes assessment, length of follow-up, and statistical analysis.[16] Studies with a fatal flaw in one or more categories were rated as having a high risk of bias.

### Data synthesis

Because data was insufficient to conduct quantitative analyses, we summarized findings qualitatively.

### Rating strength of evidence

We rated the strength of the available evidence for specific key questions and outcomes in a four-part hierarchy (high, moderate, low, and very low) using an approach proposed by the GRADE working group.[17] It incorporates four key elements: study design, study quality (risk of bias), consistency of results, and directness (availability of data on outcomes or populations of interest) and offers an estimation of the level of confidence in the estimate of effect.

## Results

**Figure 1:**
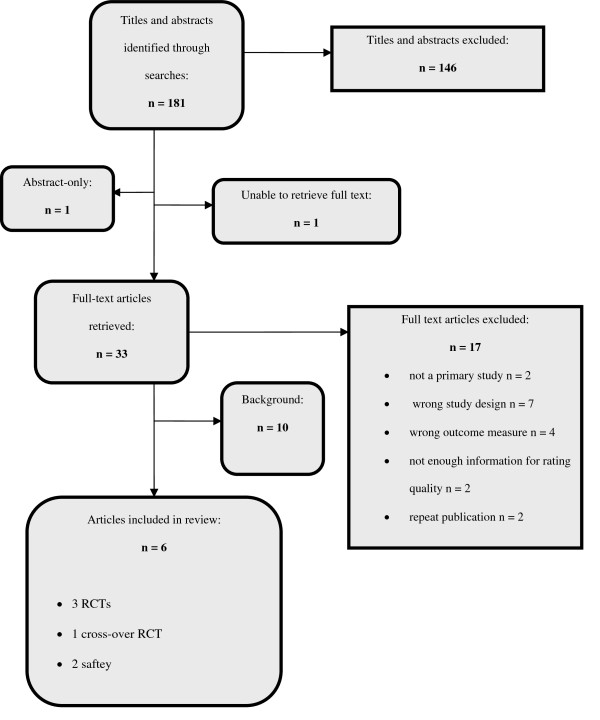
**Disposition of the literature**.

Overall we identified 181 citations. Figure 1 illustrates the disposition of the literature. We included four randomized controlled trials (RCTs) that randomized patients to BFRs or placebo for either examination anxiety [3,18,19] or ADHD.[20] We formally excluded two RCTs that were both published as letters to the editor because they contained too little information to assess their quality and we were unable to obtain the necessary information from the authors: one evaluating BFRs in anxiety in a psychiatric population and one using BFRs to treat attention deficit hyperactivity disorder (ADHD), however we summarize the results briefly. [9,21] We located a report of the preliminary findings of an observational trial of BFRs for 12 patients with moderate depression.[22] Despite multiple attempts, we were unfortunately unable to obtain a copy of the final publication that reportedly included additional results for 18 patients with major depression and therefore would have qualified for inclusion in our safety analysis.[23]

The individual study results are summarized and presented in table 3. Table 4 presents the strength of the evidence regarding efficacy and harms of BFRs for individual indications of interest. In the following paragraphs we summarize results of included studies by indication.

**Table 3:** Summary of study results for efficacy

**Author, Year, Reference number**	**Armstrong and Ernst 2001 **[18]	**Walach et. al. 2001 **[19]	**Pintov et. al. 2005 **[20]	**Halberstein et. al. 2007 **[3]
**Country**	United Kingdom	Germany	Israel	United States

**Study design**	RCT	Cross-over RCT	RCT	RCT

**Risk of bias**	High (loss to follow up 55%)	High (inadequate randomization)	High (loss to follow up 42.5%)	Low

**Number of patients**	100	61	40	111

**Loss to follow-up**	55%	9.8%	42.5%	None

**Study population**	Students with examination anxiety	Students with examination anxiety registered to take at least two exams, two weeks apart	Children with clinical diagnosis of any subtype of ADHD	Nursing students with examination anxiety

**Patient age (range and/or average, in years)**	Not reported	28	7–11	25.6 (18–49)

**Indication for BFR**	Examination anxiety	Examination anxiety	ADHD	Examination anxiety

**Intervention**	1–4 doses of Five Flower Remedy (Rescue Remedy) per day *Prunus cerasifera, Clematis vitalba, Impatiens glandulifera, Helianthemum and Ornithogalum umbellatum*	At least 4 drops of a combination of 10 BFRs per day: *Impatiens, mimulus, gentian, chestnut bud, rock rose, larch, cherry plum, white chestnut, scleranthus and elm*	4 drops of five flower BFR 4 times per day: *Prunus cerasifera, Clematis vitalba, Impatiens glandulifera, Helianthemum and Ornithogalum umbellatum*	Four drops of Rescue Remedy every 20 minutes over 3 hours: *Prunus cerasifera, Clematis vitalba, Impatiens glandulifera, Helianthemum and Ornithogalum umbellatum*

**Control**	Placebo	Placebo	Placebo	Placebo

**Duration of treatment**	7 days	4 weeks (two weeks before first exam and two weeks before second exam)	3 months	3 hours

**Main outcome measures**	State-Trait Anxiety Inventory (STAI)	German Test Anxiety Inventory (TAI-G)	Conner's scale	State-Trait Anxiety Inventory (STAI) for Adults, S-Anxiety subscale

**Results**	No significant difference in State anxiety score (on the night before examination most likely to cause anxiety) between experimental and placebo groups – 51.5 in BFR group, 54.4 in placebo group (P = 0.834)	No significant difference between experimental and placebo group for mean reduction in test anxiety: 5.25 in BFR group, 7.69 in placebo group (P = 0.55)	No significant difference in mean Conner's scores between experimental and placebo group before (16.59 in BFR group, 17.12 in placebo group) or after (11.90 in BFR group, 13.58 in placebo group) treatment (P not reported).	No significant difference in STAI S-Anxiety subscale between treatment and placebo groups.

RCT : (randomised controlled trial)

**Table 4:** Evidence profile for Bach flower remedies

**Number of studies (patients)**	**Design**	**Risk of bias**	**Consistency of results**	**Directness**	**Size of effect**	**Other modifying factors**	**Strength of the collective evidence**
**Outcome: Reduction in examination anxiety compared with control**							

3 (272)	RCT	High	Yes	Yes	Similar treatment effects and efficacy between BFR and control groups in STAI and TAI-G.	None	Low

**Outcome: Reduction in item 15 "anxiety" on VAS compared with control in psychiatric patients suffering anxiety as the main symptom**							

1 (98)	RCT	Cannot assess	N/A	Yes	No significant difference between treatment (27.40 ± 30.45) and placebo (21.73 ± 27.28)	None	Very low

**Outcome: Improvement in Connor's score compared with control (ADHD)**							

1 (40)	RCT	High	N/A	Yes	4.69 points reduction in Connor's score, no significant difference between treatment and placebo	None	Very low

**Outcome: Attenuation of stress**							

*No evidence*							

**Outcome: Depression**							

*No evidence*							

**Outcome: Pain relief**							

*No evidence*							

**Outcome: Quality of life**							

*No evidence*							

**Outcome: Adverse events**							

6 (468)	RCT, case series	High	Yes	Yes	3 experimental group subjects had side effects	None	Very low

STAI: State-Trait Anxiety Inventory, TAI-G: German version of the Test Anxiety Inventory, VAS: Visual Analogue Scale, ADHD: Attention-deficit/Hyperactivity Disorder, RCT: Randomized Controlled Trial; N/A: not applicable

Strength of the evidence (GRADE approach): High: further research is very unlikely to change our confidence in the estimate of effect; Moderate: further research is likely to have an important impact on our confidence in the estimate of effect and may change the estimate; Low: further research is very likely to have an important impact on our confidence in the estimate of effect and is likely to change the estimate; Very Low: any estimate of effect is very uncertain.

### Efficacy of BFRs for examination anxiety

Three placebo-controlled RCTs examined the efficacy of BFRs for the treatment of examination anxiety in 272 students between the ages of 18 and 65. [3,18,19] Two studies used the Five Flower Rescue Remedy, a third a combination of 10 BFRs as interventions. Study durations ranged from three hours to 14 days. Examination anxiety was measured using the State-Trait Anxiety Inventory for Adults (STAI),[18] the STAI S-Anxiety subscale,[3] or the German version of the Test Anxiety Inventory (TAI-G).[19]

The best available evidence was a RCT conducted in the United States (US) which randomized 111 nursing students to Rescue Remedy or placebo after leading them to believe they had to take a surprise examination.[3] The students applied four drops of Rescue Remedy or placebo to their tongues every 20 minutes for a period of three hours. Overall, the study was well conducted and had a low risk of bias. Both participants and study personnel were blinded to the treatment allocation, and the randomization sequence was computer generated. Measurement of the STAI S-Anxiety subscale was performed before the intervention and again 3 hours later.

Results indicated similar treatment effects of BFRs and placebo. Students treated with BFRs exhibited a reduction of 0.44 points on the STAI S-Anxiety subscale (reduction from 2.21 to 1.77), compared with a 0.38 point reduction in students on placebo (reduction from 2.34 to 1.96). This difference did not reach statistical significance and is most likely also not clinically relevant. In post hoc ANOVA analyses of various subgroups of patients, however, BFRs reduced anxiety in the subgroup of students who demonstrated high levels of anxiety at the first measurement (N = 39), more than the placebo (data not reported; p = 0.03). However, this result should be interpreted with caution because subgroup analyses are prone to chance findings due to multiple testing, or alternatively "regression to the mean" may have occurred. [24]

The other two studies, one conducted in Germany [19], the other in the United Kingdom (UK) [18] enrolled fewer participants and both had a high risk of bias (inadequate randomization [19] or high loss to follow-up [18]). Nevertheless, findings were consistent with results from the US trial. The German study randomized 61 students to a pre-determined combination of 10 BFRs (impatiens, mimulus, gentian, chestnut bud, rock rose, larch, cherry plum, white chestnut, scleranthus, and elm) or placebo in a four-week cross-over design.[19] The RCT conducted in the UK randomized 100 students to at least one week of Five Flower Rescue Remedy or placebo.[18] Measurements were taken at baseline, the night before the examination and after the examination using the STAI and the TAI-G.[18]

Both trials did not detect any differences in efficacy between BFRs and placebo for the treatment of examination anxiety. The university students in the German study actually reported a numerically greater reduction of the TAI-G in students taking placebo than in students taking BFRs.[19] The mean reduction in the BFRs group was 5.25, compared with 7.69 in the placebo group (P = 0.55). Similarly, the UK trial [18] found no significant difference in the STAI on the night before the examination between experimental and placebo groups: The score was 51.5 in the BFRs group and 54.4 in the placebo group (P = 0.834).

### Efficacy of BFRs for anxiety in psychiatric patients

A fourth RCT, also conducted in Germany, reported on the efficacy of rescue remedy for treating anxiety in 98 psychiatric patients (with diagnoses of anxiety disorder, depression or depressive symptoms during a non-acute schizoaffective psychosis) who had anxiety as their main symptom.[21] After three days of therapy, there was no difference in improvement of the item 15 "anxiety" measure on a visual analogue scale (VAS) between the BFRs and placebo group. We were unable to include this study, as not enough information regarding randomization, masking, attrition or the statistical analysis was available to evaluate its internal validity. Numerous attempts to obtain the appropriate information from the authors unfortunately failed. Nonetheless, the results are consistent with the other trials of BFRs for anxiety.

### Efficacy of BFRs for Attention-deficit Hyperactivity Disorder (ADHD)

We found two RCTs of BFRs for ADHD. One RCT conducted in Israel reported on the efficacy of five BFRs (Prunus cerasifera, Clematis vitalba, Impatiens glandulifera, Helianthemum and Ornithogalum, the components of Five Flower/Rescue Remedy[18]) in treating forty 6–11 year old children with a DSM (Diagnostic and Statistical Manual)-VI confirmed diagnosis of ADHD.[20] The children were randomized to three months of BFR or placebo. The mean Conner's score before treatment was 16.59 in the BFRs group and 17.12 in the placebo group. After treatment, the corresponding values were 11.90 and 13.58, indicating a significant improvement over time for both groups (p = 0.001), however no statistically significant difference between BFRs and placebo (p value not given). Again, the very high dropout rate (42.5%) in this study, attributed to difficulty in giving children the drops four times a day, indicates the study has a high risk of bias.

We excluded the second randomized, controlled trial of 10 children aged 5- to 12 with a diagnosis of ADHD requiring partial hospitalization, which was published in abbreviated form as a letter to the editor.[9] Not enough information regarding randomization, masking or statistical analysis was available to rate its internal validity. The children received either Rescue Remedy plus vervain, crab apple and walnut BFRs or a placebo 2–3 times a day, with follow-up performed at three weeks and three months. None of the 10 children completed the study. The authors report no difference in the Columbia Impairment Scale (CIS) score, however a significant difference between Childhood Attention Profile (CAP) score at both three weeks and three months is reported (p = 0.03; data not reported).

### Efficacy of BFRs for stress

Two randomized controlled trials from the same author examined the efficacy of BFRs in subjects under stress.[25,26] We excluded both studies, as they did not meet our pre-specified inclusion criteria, however due to lack of evidence we summarize them briefly here. One study randomized the 24 participants to rescue remedy and Yarrow Special Formula, which contains non-Bach flower essences (*Achillea millefolium*, *Achillea millefolium var. rubra*, *Achillea filipendulina*, *Arnica mollis*, *Echinacea purpurea*).[26,27] The subjects underwent "intense environmental stimulation by fluorescent lights and their accompanying electromagnetic fields". The second study randomized 24 participants aged 18–67 to Five Flower Formula (Rescue Remedy) or placebo, and submitted the participants to a Paced Serial Arithmetic Task (PSAT).[25] Stress levels were determined by quantitative electroencephalography (qEEG), surface electromyography (sEMG), or hand temperature and skin conductance. None of these parameters meet our pre-specified outcome criteria (improvement of symptoms).[28] Overall, there were no significant differences between the BFRs and placebo groups.

### Efficacy of BFRs for depression

We located one publication, titled "preliminary findings", of an open time-series that compared one month of usual care followed by three months of usual care plus individualized flower essence therapy in 12 moderately depressed patients.[22] This study was not controlled and included non-BFR essences and therefore we excluded it. Another study by the same author with the same design was included in a previous systematic review and reported as including 12 patients with moderate depression and 18 patients with major depression.[23] Based on available information the study would not have met our inclusion criteria for efficacy (not controlled, non-BFR essences). Despite multiple attempts we were unable to locate the full text to confirm the exclusion. There was no safety information reported in the preliminary findings publication.[22]

### Efficacy of BFRs for pain relief

We did not find any studies on the use of BFRs in pain that met our inclusion criteria for efficacy. We briefly summarize the findings of one retrospective case series because it is the only available evidence. [8] The study analyzed 41 case reports of patients with pain who were amongst 389 patients treated with BFRs by students of The Dr. Edward Bach Foundation's practitioner training course in England. In this study, 19 of the 41 case reports described an improvement in the patient's physical pain, and 36 of the 41 case reports reported an improvement in emotional outlook. In 20 cases there was no information regarding pain.

### Harms

Of the four controlled trials, one did not make any reference to harms of BFRs.[20] In total, only four adverse events were reported in subjects taking BFRs. [18,19] Three of the British university students in the BFR group had adverse events; headache was reported by two subjects, one withdrew from the study after three days as a result, and one subject reported skin eruptions.[18] Walach et al.[19] reported one subject with an adverse event; however they did not specify the nature of the event or whether it occurred in a subject taking BFRs or placebo. In addition, we found two observational studies of BFRs with more than 30 participants, one retrospective case series of BFRs for pain, [8] and one prospective observational study of 115 patients performed in Italy.[29] Both observational studies demonstrated a high risk of bias. The observational study of BFRs for pain did not report on adverse events.[8] None of the participants in the Italian observational study or American RCT suffered an adverse event.[3,29] The six studies included data on 468 patients. None of the trials defined adverse events in advance and all relied on participant self-reporting of adverse events. Table 5 summarizes the evidence on harms.

**Table 5:** Summary of study results for harms

**Author, Year**	**Campanini 1997**[29]	**Armstrong and Ernst 2001**[18]	**Walach et. al. 2001**[19]	**Pintov et. al. 2005**[20]	**Howard 2007**[8]	**Halberstein et. al. 2007**[3]
**Study design**	Case series	Placebo-controlled RCT	Placebo-controlled RCT	Placebo-controlled trial	Case series	RCT

**N**	115	100	61	40	41	111

**Adverse Events**	No AEs	BFRs group: 3 AEs: Headaches Skin eruptions	1 reported AE not specified	Not reported	Not reported	No AEs
		Placebo group 3 AEs: Vomiting before the examination Hayfever symptoms Depressive mood				

AEs: Adverse Events

### Recruitment and funding of included trials

The three trials that assessed the effect of BFRs in examination anxiety recruited students from university campuses. [18,19,3] One of the studies was supported by the Cecil Pilkington Trust (a British-based private charitable organisation) and a grant from "The Twelve Healers Trust" (who sponsor the "Bach Flower Research Programme" website). [18,30] The other studies [3,19] reported no commercial or private funding, although BFRs were supplied to one study by The Nelson Company.[3] The authors reporting on BFR in ADHD did not specify participant recruitment methods or funding.[20]

## Discussion

### Summary of the main findings

The evidence regarding Bach Flower Remedies for psychological problems is very limited and the majority of studies have methodological problems. Only four prospective controlled studies were available. [19,18,20,3] All of these indicated that BFRs are not effective over and above the effects of placebo for examination-related anxiety or ADHD. We could not find any controlled evidence examining the efficacy of BFRs for the treatment of pain. Nor did we find any evidence on the efficacy of BFRs for other psychological problems for which their use is suggested, such as phobias.[9]

Overall, we rated the strength of the evidence for the treatment of examination-related anxiety as low, indicating that future studies might have an important impact on the estimate of the effect. For the treatment of anxiety in psychiatric patients, and ADHD in children, as well as for the risk of harms we deemed the strength of the evidence to be very low, indicating that any estimate of the effect is very uncertain.

### Strengths and limitations of this review

We evaluated the evidence for BFR using the standard methodology of evidence-based medicine (EBM). Thus, we rated five of the six included trials as being at high risk of bias. We recognise that the evaluation of complementary and alternative medicine practices using EBM principles is controversial. [31,32] Although proponents of EBM point out that even interventional forms of CAM (e.g., acupuncture) have successfully incorporated randomisation and blinding into their study design and evaluation,[31] and that an individualized approach to therapy does not preclude blinding or randomisation, some CAM researchers fear that focussing exclusively on the internal validity of trials means ignoring important non-quantifiable differences between individuals, and leads to conclusions that do not represent the effectiveness of treatments under real-world conditions.[32] Moreover, the emphasis on self-healing and self-awareness integral to CAM (and Bach flower therapy as described by Bach) is not able to be quantified, and hence not able to be assessed.[32] CAM practitioners have argued that the process of defining specific outcomes, randomising patients to placebos, and blinding both therapist and patient interferes with the holistic and individualised practitioner-patient relationship and produces unreliable and unfair results that do not reflect the true use of the therapy in its original form.[32] For example, one Bach flower practitioner argues that "qualities such as compassion, trust, empathy, and positive motivation can directly help to improve outcomes".[8] Indeed, a recent survey of physicians demonstrated that many prescribe inert or minimally active pharmacological substances, presumably in order to "promote positive therapeutic expectations".[33]

All of the studies in this review used a pre-determined mixture of BFRs which does not correspond with their use in real practice. Furthermore, the anxiety states in several of the trials were experimentally induced and may differ from typical anxiety as experienced by patients. The short period of BFR administration in several of the trials (ten minutes, three hours, one week) does not comply with the recommendations of BFR practitioners, though it may be typical of "over-the-counter" use.[4]

Because RCTs are often criticized as being unsuitable for assessing CAM interventions, we specifically included not only RCTs, but also non-randomized controlled trails and large (N ≥ 30) observational studies in our review. Despite this, we were able to include only four trials and two observational studies. Because the evidence was insufficient to be pooled we did not conduct any formal statistical tests such as funnel plots or Kendell's test to assess publication bias. We searched clinical trials registries and could not detect any studies that were registered but have not been published. Nevertheless, publication bias is always major threat to systematic reviews. Similarly, retrieval bias cannot be ruled out.

### Comparison with existing literature

This is, to our knowledge, the first systematic review of BFRs that has followed the QUORUM recommendations (Quality of Reporting of Meta-analyses).[34] A systematic review of BFRs published in 2002 concluded that the four available studies did not indicate that BFRs are clinically different to placebo for overdue birth, examination anxiety and depression.[6] Our findings are consistent with this review. In addition, we located several additional studies that failed to demonstrate a benefit beyond the placebo effect in examination anxiety and ADHD and we provide the first summary of the evidence for the safety of BFRs.

## Conclusion

Our review demonstrates that the currently available evidence indicates that BFRs are not more efficacious than a placebo intervention for psychological problems but are probably safe. Due to a lack of methodologically sound trials, this statement is associated with a high level of uncertainty. Recognising the need for evaluation of various CAM practices, the US National Institute of Health now provides over 121 million dollars of funding annually for evidence-based research into CAM via the National Centre for Complementary and Alternative Medicine (NCCAM).[35] We recommend that future trials employ methods that minimize bias and confounding such as randomization and blinding; that trials are conducted with an adequate sample size to be able to detect effects with statistical significance; that attempts are made to minimize loss-to-follow-up; and that patient-relevant end-point parameters are analyzed.[28] Furthermore, we think it appropriate and important for the applicability of the evidence that adequate flexibility in personalising BFR therapy and a suitable length of follow-up is allowed. We look forward to this future good quality research that will contribute to the evidence we have presented.

## Competing interests

The authors declare that they have no competing interests.

## Authors' contributions

KT coordinated the review, applied inclusion criteria, extracted data and drafted the manuscript. AK applied inclusion criteria, extracted data, and contributed to the manuscript. AC performed the literature search and edited the manuscript. TL applied inclusion criteria, extracted data and was involved in the initial conception of the review. GG participated in the design of the review, gave methodological advice, and edited the manuscript. All authors read and approved the final manuscript.

## Pre-publication history

The pre-publication history for this paper can be accessed here:



## Supplementary Material

Additional file 1 **Basic search strategy and terms used. This was adapted depending on the database used**. This file provides a more detailed description of the search strategy and search terms. Click here for file
