# Supplementary material for: Bach Flower Remedies for psychological problems and pain: a systematic review
Source: BMC Complement Altern Med. 2009 May 26;9:16. doi: 10.1186/1472-6882-9-16 (PMC2695424; doi:10.1186/1472-6882-9-16)
Supplement: Additional file 1 — Basic search strategy and terms used. This was adapted depending on the database used. This file provides a more detailed description of the search strategy and search terms. [file 1472-6882-9-16-S1.doc]

**Additional file 1.**

**Online Search**

Date of search, 12 June 2008

Search strategy used for Medline search

| [#1](http://www.ncbi.nlm.nih.gov.libproxy.lib.unc.edu/sites/?querykey=1&dbase=pubmed&tab=History&) | flower remed* | [14](http://www.ncbi.nlm.nih.gov.libproxy.lib.unc.edu/sites/?cmd=HistorySearch&querykey=1&tab=&) |
| --- | --- | --- |
| [#2](http://www.ncbi.nlm.nih.gov.libproxy.lib.unc.edu/sites/?querykey=2&dbase=pubmed&tab=History&) | flower therap* | [7](http://www.ncbi.nlm.nih.gov.libproxy.lib.unc.edu/sites/?cmd=HistorySearch&querykey=2&tab=&) |
| [#3](http://www.ncbi.nlm.nih.gov.libproxy.lib.unc.edu/sites/?querykey=3&dbase=pubmed&tab=History&) | "rescue remedy" | [2](http://www.ncbi.nlm.nih.gov.libproxy.lib.unc.edu/sites/?cmd=HistorySearch&querykey=3&tab=&) |
| [#4](http://www.ncbi.nlm.nih.gov.libproxy.lib.unc.edu/sites/?querykey=4&dbase=pubmed&tab=History&) | bach flower* | [17](http://www.ncbi.nlm.nih.gov.libproxy.lib.unc.edu/sites/?cmd=HistorySearch&querykey=4&tab=&) |
| [#5](http://www.ncbi.nlm.nih.gov.libproxy.lib.unc.edu/sites/?querykey=5&dbase=pubmed&tab=History&) | flower essenc* | [8](http://www.ncbi.nlm.nih.gov.libproxy.lib.unc.edu/sites/?cmd=HistorySearch&querykey=5&tab=&) |
| [#6](http://www.ncbi.nlm.nih.gov.libproxy.lib.unc.edu/sites/?querykey=6&dbase=pubmed&tab=History&) | #1 OR #2 OR #3 OR #4 OR #5 | [32](http://www.ncbi.nlm.nih.gov.libproxy.lib.unc.edu/sites/?cmd=HistorySearch&querykey=6&tab=&) |

Search strategy used for The Cochrane Library search

| [#1](http://www.ncbi.nlm.nih.gov.libproxy.lib.unc.edu/sites/?querykey=1&dbase=pubmed&tab=History&) | [flower NEXT remed*](http://www3.interscience.wiley.com/cochrane/searchHistory?mode=runquery&qnum=1) | 5 |
| --- | --- | --- |
| [#2](http://www.ncbi.nlm.nih.gov.libproxy.lib.unc.edu/sites/?querykey=2&dbase=pubmed&tab=History&) | [flower NEXT therap*](http://www3.interscience.wiley.com/cochrane/searchHistory?mode=runquery&qnum=2) | 1 |
| [#3](http://www.ncbi.nlm.nih.gov.libproxy.lib.unc.edu/sites/?querykey=3&dbase=pubmed&tab=History&) | [rescue NEXT remed*](http://www3.interscience.wiley.com/cochrane/searchHistory?mode=runquery&qnum=3) | 1 |
| [#4](http://www.ncbi.nlm.nih.gov.libproxy.lib.unc.edu/sites/?querykey=4&dbase=pubmed&tab=History&) | [bach NEXT flower*](http://www3.interscience.wiley.com/cochrane/searchHistory?mode=runquery&qnum=4) | 6 |
| [#5](http://www.ncbi.nlm.nih.gov.libproxy.lib.unc.edu/sites/?querykey=5&dbase=pubmed&tab=History&) | [flower NEXT essenc*](http://www3.interscience.wiley.com/cochrane/searchHistory?mode=runquery&qnum=5) | 2 |
| [#6](http://www.ncbi.nlm.nih.gov.libproxy.lib.unc.edu/sites/?querykey=6&dbase=pubmed&tab=History&) | #1 OR #2 OR #3 OR #4 OR #5 | [1](http://www.ncbi.nlm.nih.gov.libproxy.lib.unc.edu/sites/?cmd=HistorySearch&querykey=6&tab=&)1 |

Search strategy used for Embase search

| [#1](http://www.ncbi.nlm.nih.gov.libproxy.lib.unc.edu/sites/?querykey=1&dbase=pubmed&tab=History&) | flower:ti AND essenc*:ti OR bach:ti AND flower*:ti OR rescue:ti OR rescue:ti AND remed*:ti OR flower:ti AND therap*:ti OR flower:ti AND remed*:ti | 16 |
| --- | --- | --- |
| [#2](http://www.ncbi.nlm.nih.gov.libproxy.lib.unc.edu/sites/?querykey=2&dbase=pubmed&tab=History&) | ti:ab AND flower:ab AND essenc*:ab OR bach:ab AND flower*:ab OR rescue:ab OR rescue:ab AND remed*:ab OR flower:ab AND therap*:ab OR flower:ab AND remed*:ab | 38 |
| [#3](http://www.ncbi.nlm.nih.gov.libproxy.lib.unc.edu/sites/?querykey=3&dbase=pubmed&tab=History&) | [rescue NEXT remed*](http://www3.interscience.wiley.com/cochrane/searchHistory?mode=runquery&qnum=3) | 45 |

Search strategy used for AMED search

| [#1](http://www.ncbi.nlm.nih.gov.libproxy.lib.unc.edu/sites/?querykey=1&dbase=pubmed&tab=History&) | flower remed* |  |
| --- | --- | --- |
| [#2](http://www.ncbi.nlm.nih.gov.libproxy.lib.unc.edu/sites/?querykey=2&dbase=pubmed&tab=History&) | flower therap* |  |
| [#3](http://www.ncbi.nlm.nih.gov.libproxy.lib.unc.edu/sites/?querykey=3&dbase=pubmed&tab=History&) | "rescue remedy" |  |
| [#4](http://www.ncbi.nlm.nih.gov.libproxy.lib.unc.edu/sites/?querykey=4&dbase=pubmed&tab=History&) | bach flower* |  |
| [#5](http://www.ncbi.nlm.nih.gov.libproxy.lib.unc.edu/sites/?querykey=5&dbase=pubmed&tab=History&) | flower essenc* |  |
| [#6](http://www.ncbi.nlm.nih.gov.libproxy.lib.unc.edu/sites/?querykey=6&dbase=pubmed&tab=History&) | #1 OR #2 OR #3 OR #4 OR #5 | [100](http://www.ncbi.nlm.nih.gov.libproxy.lib.unc.edu/sites/?cmd=HistorySearch&querykey=6&tab=&) |

**Hand search**

Date of search, from 11 August 2008 to 13 August 2008.

We manually searched the reference lists of all 7 articles included in the review.

**Contact**

We contacted the following organizations:

- the Bach flower research programme, www.edwardbach.org
- BioMed Central, www.biomedcentral.com
- The International Society for the Study of Subtle Energies and Energy Medicine (ISSSEEM), http://www.issseem.org/
- CAM Magazine, www.cam-mag.com

We contacted the following people:

Edzard Ernst MD, PhD, FRCP, FRCPEd, Edzard.Ernst@pms.ac.uk
